# *N*-(3-Chloro-4-fluoro­phen­yl)-2-(naphthalen-1-yl)acetamide

**DOI:** 10.1107/S1600536811024597

**Published:** 2011-06-25

**Authors:** A. S. Praveen, Jerry P. Jasinski, James A. Golen, B. Narayana, H. S. Yathirajan

**Affiliations:** a Department of Studies in Chemistry, University of Mysore, Manasagangotri, Mysore 570 006, India; b Department of Chemistry, Keene State College, 229 Main Street, Keene, NH 03435-2001, USA; c Department of Studies in Chemistry, Mangalore University, Mangalagangotri 574 199, India

## Abstract

In the title compound, C_18_H_13_ClFNO, the dihedral angle between the mean planes of the chloro- and fluoro-substituted benzene ring and the naphthalene ring system is 60.5 (8)°. In the crystal, mol­ecules are linked by N—H⋯O hydrogen bonds, forming a zigzag chain along [101].

## Related literature

For the structural similarity of *N*-substituted 2-aryl­acetamides to the lateral chain of natural benzyl­penicillin, see: Mijin & Marinkovic (2006 ▶); Mijin *et al.* (2008 ▶). For the coordination abilities of amides, see: Wu *et al.* (2008 ▶, 2010 ▶). For related structures, see: Davis & Healy (2010 ▶); Li *et al.* (2010 ▶); Li & Wu (2010 ▶); Wang *et al.* (2010 ▶); Xiao *et al.* (2010 ▶). For standard bond lengths, see: Allen *et al.* (1987 ▶).
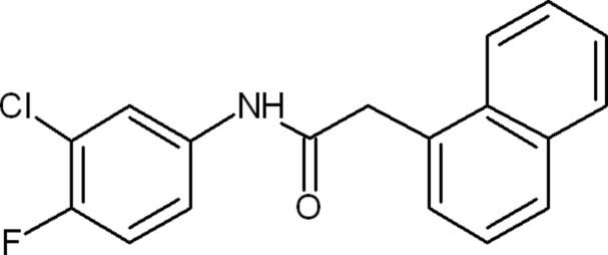


## Experimental

### 

#### Crystal data

C_18_H_13_ClFNO
*M* *_r_* = 313.74
Monoclinic, 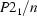

*a* = 8.096 (6) Å
*b* = 23.323 (6) Å
*c* = 8.404 (3) Å
β = 110.83 (5)°
*V* = 1483.4 (13) Å^3^
*Z* = 4
Mo *K*α radiation
μ = 0.27 mm^−1^
*T* = 173 K
0.30 × 0.18 × 0.10 mm

#### Data collection

Oxford Diffraction Oxford Xcalibur Eos Gemini diffractometer
Absorption correction: multi-scan (*CrysAlis RED*; Oxford Diffraction, 2010 ▶) *T*_min_ = 0.924, *T*_max_ = 0.974
13979 measured reflections
3679 independent reflections
2947 reflections with *I* > 2σ(*I*)
*R*_int_ = 0.024

#### Refinement

*R*[*F* ^2^ > 2σ(*F* ^2^)] = 0.044
*wR*(*F* ^2^) = 0.119
*S* = 1.04
3679 reflections
202 parameters
1 restraint
H atoms treated by a mixture of independent and constrained refinement
Δρ_max_ = 0.25 e Å^−3^
Δρ_min_ = −0.31 e Å^−3^

### 

Data collection: *CrysAlis PRO* (Oxford Diffraction, 2010 ▶); cell refinement: *CrysAlis PRO*; data reduction: *CrysAlis RED* (Oxford Diffraction, 2010 ▶); program(s) used to solve structure: *SHELXS97* (Sheldrick, 2008 ▶); program(s) used to refine structure: *SHELXL97* (Sheldrick, 2008 ▶); molecular graphics: *SHELXTL* (Sheldrick, 2008 ▶); software used to prepare material for publication: *SHELXTL*.

## Supplementary Material

Crystal structure: contains datablock(s) global, I. DOI: 10.1107/S1600536811024597/is2737sup1.cif

Structure factors: contains datablock(s) I. DOI: 10.1107/S1600536811024597/is2737Isup2.hkl

Supplementary material file. DOI: 10.1107/S1600536811024597/is2737Isup3.cml

Additional supplementary materials:  crystallographic information; 3D view; checkCIF report

## Figures and Tables

**Table 1:** Hydrogen-bond geometry (Å, °)

*D*—H⋯*A*	*D*—H	H⋯*A*	*D*⋯*A*	*D*—H⋯*A*
N1—H1*N*⋯O1^i^	0.85 (1)	2.12 (2)	2.914 (2)	157 (2)

Symmetry code: (i) 
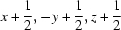
.

**Fig. 1.**
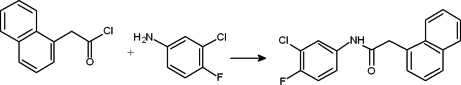
Reaction scheme of the title compound, (I).

**Fig. 2.**
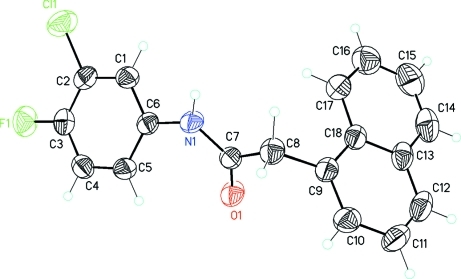
Molecular structure of the title compound, showing the atom labeling scheme and 50% probability displacement ellipsoids.

**Fig. 3.**
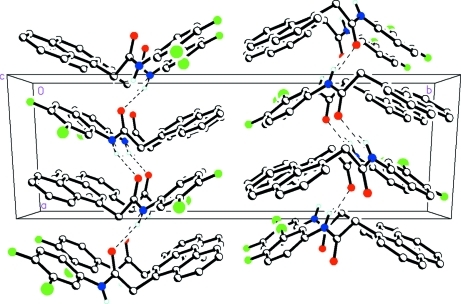
Packing diagram of the title compound viewed down the c axis. Dashed lines represent N—H···O hydrogen bonds.

## supplementary crystallographic information

### Comment

N-Substituted 2-arylacetamides are very interesting compounds because of their structural similarity to the lateral chain of natural benzylpenicillin (Mijin *et al.*, 2006, 2008). Amides are also used as ligands due to their excellent coordination abilities (Wu *et al.*, 2008, 2010).  Crystal structures of some acetamide derivatives, viz., 2-(4-bromophenyl)-N-(2-methoxyphenyl)acetamide (Xiao *et al.*, 2010), N-benzyl-2-(3-chloro-4-hydroxyphenyl)acetamide (Davis & Healy, 2010), 2-(2,2-dimethyl-2,3-dihydro-1-benzofuran-7-yloxy)-N-(o-tolyl)acetamide (Li *et al.*, 2010), N-benzyl-2-(2-bromophenyl)-2-(2-nitrophenoxy) acetamide (Li & Wu, 2010) and N-(4-chlorophenyl)-2-(8-quinolyloxy)acetamide monohydrate (Wang *et al.*, 2010) have been reported. In view of the importance of amides, we report herein the crystal structure of the title compound, (I), C_18_H_13_ClFNO.

In the title compound, C_18_H_13_ClFNO, the dihedral angle between the mean planes of the chloro, fluoro substituted benzene ring and the naphthalene-1-yl ring is 60.5 (8)° (Fig. 2).  Bond distances are in normal ranges (Allen *et al.*, 1987). Crystal packing is stabilized by N—H···O hydrogen bonds (Fig. 3 and Table 1).

### Experimental

Naphthalen-1-ylacetyl chloride (0.204 g, 1 mmol) and 3-chloro-4-fluoroaniline (0.145 g, 1 mmol) were dissolved in dichloromethane (20 mL). The mixture was stirred in presence of triethylamine at 273 K for about 3 h (Fig. 1).  The contents were poured into 100 ml of ice-cold aqueous hydrochloric acid with stirring, which was extracted thrice with dichloromethane. Organic layer was washed with saturated NaHCO_3_ solution and brine solution, dried and concentrated under reduced pressure to give the title compound (I).  Single crystals were grown from toluene by the slow evaporation method (M.P.: 421 K).

### Refinement

The N-bound H atom was located in a difference Fourier map and refined isotropically with a distance restraint of N—H = 0.86 (2) Å. All of the remaining H atoms were placed in their calculated positions and then refined using the riding model, with C—H lengths of 0.95 Å (CH) or 0.99 Å (CH_2_). Isotropic displacement parameters for these atoms were set to 1.19-1.21 (CH) or 1.20 (CH_2_) times *U*_eq_ of the parent atom.

### Crystal data

**Table:**

C_18_H_13_ClFNO	*F*(000) = 648
*M**_r_* = 313.74	*D*_x_ = 1.405 Mg m^−^^3^
Monoclinic, *P*2_1_/*n*	Mo *K*α radiation, λ = 0.71073 Å
Hall symbol: -P 2yn	Cell parameters from 4655 reflections
*a* = 8.096 (6) Å	θ = 3.1–32.5°
*b* = 23.323 (6) Å	µ = 0.27 mm^−^^1^
*c* = 8.404 (3) Å	*T* = 173 K
β = 110.83 (5)°	Block, colorless
*V* = 1483.4 (13) Å^3^	0.30 × 0.18 × 0.10 mm
*Z* = 4	

### Data collection

**Table:**

Oxford Diffraction Oxford Xcalibur Eos Gemini diffractometer	3679 independent reflections
Radiation source: Enhance (Mo) X-ray Source	2947 reflections with *I* > 2σ(*I*)
graphite	*R*_int_ = 0.024
Detector resolution: 16.1500 pixels mm^-1^	θ_max_ = 28.3°, θ_min_ = 3.1°
ω scans	*h* = −10→10
Absorption correction: multi-scan (*CrysAlis RED*; Oxford Diffraction, 2010)	*k* = −31→31
*T*_min_ = 0.924, *T*_max_ = 0.974	*l* = −10→11
13979 measured reflections	

### Refinement

**Table:**

Refinement on *F*^2^	Primary atom site location: structure-invariant direct methods
Least-squares matrix: full	Secondary atom site location: difference Fourier map
*R*[*F*^2^ > 2σ(*F*^2^)] = 0.044	Hydrogen site location: inferred from neighbouring sites
*wR*(*F*^2^) = 0.119	H atoms treated by a mixture of independent and constrained refinement
*S* = 1.04	*w* = 1/[σ^2^(*F*_o_^2^) + (0.0495*P*)^2^ + 0.4725*P*] where *P* = (*F*_o_^2^ + 2*F*_c_^2^)/3
3679 reflections	(Δ/σ)_max_ = 0.001
202 parameters	Δρ_max_ = 0.25 e Å^−^^3^
1 restraint	Δρ_min_ = −0.31 e Å^−^^3^

### Special details

**Table:**

Geometry. All esds (except the esd in the dihedral angle between two l.s. planes) are estimated using the full covariance matrix. The cell esds are taken into account individually in the estimation of esds in distances, angles and torsion angles; correlations between esds in cell parameters are only used when they are defined by crystal symmetry. An approximate (isotropic) treatment of cell esds is used for estimating esds involving l.s. planes.
Refinement. Refinement of *F*^2^ against ALL reflections. The weighted *R*-factor *wR* and goodness of fit *S* are based on *F*^2^, conventional *R*-factors *R* are based on *F*, with *F* set to zero for negative *F*^2^. The threshold expression of *F*^2^ > σ(*F*^2^) is used only for calculating *R*-factors(gt) *etc*. and is not relevant to the choice of reflections for refinement. *R*-factors based on *F*^2^ are statistically about twice as large as those based on *F*, and *R*- factors based on ALL data will be even larger.

### Fractional atomic coordinates and isotropic or equivalent isotropic displacement parameters (Å^2^)

**Table:**

	*x*	*y*	*z*	*U*_iso_*/*U*_eq_	
Cl1	0.38936 (7)	0.11723 (2)	1.00561 (6)	0.06507 (18)	
F1	0.17103 (16)	0.03432 (5)	0.75440 (15)	0.0629 (3)	
O1	0.21705 (14)	0.22471 (5)	0.22432 (14)	0.0486 (3)	
N1	0.43807 (16)	0.20845 (6)	0.47811 (16)	0.0379 (3)	
H1N	0.538 (2)	0.2204 (7)	0.544 (2)	0.046*	
C1	0.4025 (2)	0.16306 (7)	0.71760 (19)	0.0380 (3)	
H1B	0.4762	0.1917	0.7880	0.046*	
C2	0.3379 (2)	0.11924 (7)	0.7882 (2)	0.0414 (4)	
C3	0.2317 (2)	0.07766 (7)	0.6847 (2)	0.0428 (4)	
C4	0.1869 (2)	0.07943 (7)	0.5115 (2)	0.0426 (4)	
H4A	0.1126	0.0507	0.4417	0.051*	
C5	0.2506 (2)	0.12309 (6)	0.43962 (19)	0.0385 (3)	
H5A	0.2205	0.1247	0.3197	0.046*	
C6	0.35908 (18)	0.16496 (6)	0.54278 (18)	0.0346 (3)	
C7	0.36895 (18)	0.23348 (6)	0.32430 (18)	0.0347 (3)	
C8	0.4966 (2)	0.27172 (7)	0.2798 (2)	0.0407 (3)	
H8A	0.5850	0.2870	0.3858	0.049*	
H8B	0.5604	0.2488	0.2209	0.049*	
C9	0.40433 (19)	0.32093 (6)	0.16728 (19)	0.0373 (3)	
C10	0.3683 (2)	0.31871 (8)	−0.0039 (2)	0.0460 (4)	
H10A	0.4071	0.2866	−0.0507	0.055*	
C11	0.2745 (2)	0.36315 (9)	−0.1131 (2)	0.0555 (5)	
H11A	0.2509	0.3606	−0.2320	0.067*	
C12	0.2185 (2)	0.40899 (8)	−0.0499 (2)	0.0545 (5)	
H12A	0.1538	0.4383	−0.1248	0.065*	
C13	0.2547 (2)	0.41419 (7)	0.1263 (2)	0.0449 (4)	
C14	0.2016 (3)	0.46210 (8)	0.1974 (3)	0.0609 (5)	
H14A	0.1363	0.4918	0.1247	0.073*	
C15	0.2418 (3)	0.46677 (9)	0.3675 (3)	0.0695 (6)	
H15A	0.2056	0.4997	0.4130	0.083*	
C16	0.3361 (3)	0.42340 (9)	0.4765 (3)	0.0633 (5)	
H16A	0.3648	0.4274	0.5958	0.076*	
C17	0.3873 (2)	0.37576 (7)	0.4146 (2)	0.0482 (4)	
H17A	0.4493	0.3463	0.4906	0.058*	
C18	0.34938 (19)	0.36936 (7)	0.2373 (2)	0.0384 (3)	

### Atomic displacement parameters (Å^2^)

**Table:**

	*U*^11^	*U*^22^	*U*^33^	*U*^12^	*U*^13^	*U*^23^
Cl1	0.0760 (4)	0.0795 (4)	0.0392 (2)	−0.0043 (3)	0.0199 (2)	0.0062 (2)
F1	0.0707 (7)	0.0521 (6)	0.0693 (7)	−0.0078 (5)	0.0291 (6)	0.0142 (5)
O1	0.0360 (6)	0.0473 (6)	0.0456 (6)	−0.0067 (5)	−0.0064 (5)	0.0111 (5)
N1	0.0282 (6)	0.0420 (7)	0.0344 (6)	−0.0039 (5)	−0.0002 (5)	0.0035 (5)
C1	0.0342 (7)	0.0390 (8)	0.0366 (7)	0.0023 (6)	0.0072 (6)	−0.0021 (6)
C2	0.0395 (8)	0.0478 (9)	0.0359 (7)	0.0078 (7)	0.0120 (6)	0.0047 (6)
C3	0.0392 (8)	0.0380 (8)	0.0522 (9)	0.0042 (6)	0.0175 (7)	0.0077 (7)
C4	0.0385 (8)	0.0351 (7)	0.0492 (9)	−0.0008 (6)	0.0096 (7)	−0.0033 (6)
C5	0.0363 (8)	0.0395 (8)	0.0353 (7)	0.0004 (6)	0.0072 (6)	−0.0018 (6)
C6	0.0273 (6)	0.0363 (7)	0.0358 (7)	0.0044 (5)	0.0060 (6)	0.0029 (6)
C7	0.0307 (7)	0.0316 (7)	0.0352 (7)	0.0031 (5)	0.0037 (6)	0.0001 (5)
C8	0.0315 (7)	0.0425 (8)	0.0433 (8)	0.0013 (6)	0.0073 (6)	0.0056 (6)
C9	0.0296 (7)	0.0401 (8)	0.0374 (7)	−0.0049 (6)	0.0058 (6)	0.0060 (6)
C10	0.0420 (9)	0.0526 (10)	0.0402 (8)	−0.0100 (7)	0.0107 (7)	−0.0010 (7)
C11	0.0505 (10)	0.0740 (13)	0.0348 (8)	−0.0145 (9)	0.0064 (7)	0.0117 (8)
C12	0.0429 (9)	0.0570 (11)	0.0527 (10)	−0.0052 (8)	0.0037 (8)	0.0241 (8)
C13	0.0333 (8)	0.0420 (8)	0.0544 (9)	−0.0035 (6)	0.0094 (7)	0.0135 (7)
C14	0.0519 (11)	0.0419 (9)	0.0868 (15)	0.0050 (8)	0.0222 (10)	0.0154 (9)
C15	0.0762 (14)	0.0506 (11)	0.0893 (16)	0.0019 (10)	0.0386 (13)	−0.0080 (11)
C16	0.0754 (14)	0.0589 (12)	0.0604 (12)	−0.0043 (10)	0.0302 (11)	−0.0072 (9)
C17	0.0509 (10)	0.0481 (9)	0.0428 (9)	−0.0026 (7)	0.0133 (8)	0.0034 (7)
C18	0.0309 (7)	0.0390 (8)	0.0414 (8)	−0.0050 (6)	0.0081 (6)	0.0063 (6)

### Geometric parameters (Å, °)

**Table:**

Cl1—C2	1.7242 (17)	C9—C10	1.363 (2)
F1—C3	1.3454 (19)	C9—C18	1.416 (2)
O1—C7	1.235 (2)	C10—C11	1.414 (3)
N1—C7	1.3457 (19)	C10—H10A	0.9500
N1—C6	1.407 (2)	C11—C12	1.343 (3)
N1—H1N	0.849 (14)	C11—H11A	0.9500
C1—C2	1.375 (2)	C12—C13	1.409 (3)
C1—C6	1.385 (2)	C12—H12A	0.9500
C1—H1B	0.9500	C13—C14	1.405 (3)
C2—C3	1.380 (2)	C13—C18	1.429 (2)
C3—C4	1.370 (2)	C14—C15	1.353 (3)
C4—C5	1.375 (2)	C14—H14A	0.9500
C4—H4A	0.9500	C15—C16	1.396 (3)
C5—C6	1.391 (2)	C15—H15A	0.9500
C5—H5A	0.9500	C16—C17	1.353 (3)
C7—C8	1.510 (2)	C16—H16A	0.9500
C8—C9	1.506 (2)	C17—C18	1.418 (2)
C8—H8A	0.9900	C17—H17A	0.9500
C8—H8B	0.9900		
			
C7—N1—C6	126.36 (13)	C10—C9—C18	119.18 (14)
C7—N1—H1N	117.4 (12)	C10—C9—C8	120.38 (15)
C6—N1—H1N	116.2 (12)	C18—C9—C8	120.41 (14)
C2—C1—C6	119.32 (14)	C9—C10—C11	121.43 (18)
C2—C1—H1B	120.3	C9—C10—H10A	119.3
C6—C1—H1B	120.3	C11—C10—H10A	119.3
C1—C2—C3	119.85 (15)	C12—C11—C10	120.37 (17)
C1—C2—Cl1	119.47 (13)	C12—C11—H11A	119.8
C3—C2—Cl1	120.68 (13)	C10—C11—H11A	119.8
F1—C3—C4	119.05 (15)	C11—C12—C13	120.77 (16)
F1—C3—C2	119.71 (15)	C11—C12—H12A	119.6
C4—C3—C2	121.24 (15)	C13—C12—H12A	119.6
C3—C4—C5	119.37 (15)	C14—C13—C12	122.34 (17)
C3—C4—H4A	120.3	C14—C13—C18	118.60 (17)
C5—C4—H4A	120.3	C12—C13—C18	119.06 (17)
C4—C5—C6	119.90 (14)	C15—C14—C13	121.24 (18)
C4—C5—H5A	120.0	C15—C14—H14A	119.4
C6—C5—H5A	120.0	C13—C14—H14A	119.4
C1—C6—C5	120.32 (14)	C14—C15—C16	120.3 (2)
C1—C6—N1	117.00 (13)	C14—C15—H15A	119.8
C5—C6—N1	122.56 (14)	C16—C15—H15A	119.8
O1—C7—N1	123.71 (15)	C17—C16—C15	120.9 (2)
O1—C7—C8	122.22 (14)	C17—C16—H16A	119.5
N1—C7—C8	114.01 (13)	C15—C16—H16A	119.5
C9—C8—C7	112.03 (13)	C16—C17—C18	120.59 (17)
C9—C8—H8A	109.2	C16—C17—H17A	119.7
C7—C8—H8A	109.2	C18—C17—H17A	119.7
C9—C8—H8B	109.2	C9—C18—C17	122.51 (14)
C7—C8—H8B	109.2	C9—C18—C13	119.17 (15)
H8A—C8—H8B	107.9	C17—C18—C13	118.31 (16)
			
C6—C1—C2—C3	0.3 (2)	C18—C9—C10—C11	−1.2 (2)
C6—C1—C2—Cl1	−179.17 (11)	C8—C9—C10—C11	176.99 (15)
C1—C2—C3—F1	178.93 (14)	C9—C10—C11—C12	0.1 (3)
Cl1—C2—C3—F1	−1.6 (2)	C10—C11—C12—C13	1.1 (3)
C1—C2—C3—C4	−0.8 (2)	C11—C12—C13—C14	178.56 (17)
Cl1—C2—C3—C4	178.65 (13)	C11—C12—C13—C18	−1.1 (2)
F1—C3—C4—C5	−179.02 (14)	C12—C13—C14—C15	−178.31 (18)
C2—C3—C4—C5	0.7 (2)	C18—C13—C14—C15	1.4 (3)
C3—C4—C5—C6	−0.1 (2)	C13—C14—C15—C16	−0.6 (3)
C2—C1—C6—C5	0.3 (2)	C14—C15—C16—C17	−0.8 (3)
C2—C1—C6—N1	−175.86 (14)	C15—C16—C17—C18	1.3 (3)
C4—C5—C6—C1	−0.4 (2)	C10—C9—C18—C17	−177.71 (15)
C4—C5—C6—N1	175.56 (14)	C8—C9—C18—C17	4.1 (2)
C7—N1—C6—C1	−150.76 (15)	C10—C9—C18—C13	1.2 (2)
C7—N1—C6—C5	33.2 (2)	C8—C9—C18—C13	−177.05 (13)
C6—N1—C7—O1	6.9 (3)	C16—C17—C18—C9	178.35 (17)
C6—N1—C7—C8	−170.51 (14)	C16—C17—C18—C13	−0.5 (2)
O1—C7—C8—C9	33.6 (2)	C14—C13—C18—C9	−179.71 (15)
N1—C7—C8—C9	−148.94 (14)	C12—C13—C18—C9	0.0 (2)
C7—C8—C9—C10	−100.15 (18)	C14—C13—C18—C17	−0.8 (2)
C7—C8—C9—C18	78.05 (18)	C12—C13—C18—C17	178.91 (15)

### Hydrogen-bond geometry (Å, °)

**Table:**

*D*—H···*A*	*D*—H	H···*A*	*D*···*A*	*D*—H···*A*
N1—H1N···O1^i^	0.85 (1)	2.12 (2)	2.914 (2)	157.(2)

Symmetry codes: (i) *x*+1/2, −*y*+1/2, *z*+1/2.
